# Short-Term Postoperative Depression and Anxiety in Patients with Differentiated Thyroid Carcinoma: Assessment of Potential Oncologic-Psycho Relevance

**DOI:** 10.1155/2024/1717119

**Published:** 2024-10-03

**Authors:** Lin Chen, Ningning Ren, Qing Yang, Xingsong Tian

**Affiliations:** ^1^Shandong University, Cheeloo College of Medicine, Jinan, Shandong, China; ^2^The Second Hospital of Shandong University, Jinan, Shandong, China; ^3^Department of Breast and Thyroid Surgery, Shandong Provincial Hospital, Jinan, Shandong, China; ^4^Department of Breast Surgery, The Second Affiliated Hospital of Zhengzhou University, Zhengzhou, Henan, China

## Abstract

*Objective*: To understand whether TSH suppressive therapy affect short-term postoperative cancer-related depression and anxiety among DTC patients. To evaluate short-term postoperative psychological problems and its relationship with baseline parameters, fatigue, sleep quality, illness perception, and patients' quality of life. *Study Design and Methods*: This was a prospective, observational, single center study. This study involved 831 TC patients who consecutively admitted to the inpatient department of hospital between 1^st^ June 2020 and 31^th^ February 2021. *Results*: Mean scores of the self-rated anxiety scale (SAS) (49.04 vs. 40.69) and self-rated depression scale (SDS) (44.61 vs. 39.86), as well as the incidence of anxiety (41.5% vs. 22.1%) and depression (22.5% vs. 2.4%) significantly decreased 3 months after surgery. For personal and clinical characteristics, low educational background (SAS, *β* = 1.392; SDS, *β* = 1.622; and *p* < 0.05), without children (SAS, *β* = 4.068; SDS, *β* = 1.873, and *p* < 0.01), FNAC (SAS, *β* = −0.981; SDS, *β* = −2.583; and *p* < 0.05), and multifocal tumor (SAS, *β* = −1.287; SDS, *β* = −2.681; and *p* < 0.05) were the main effects for both short-term postoperative anxiety and depression. Multiple linear regression analysis identified the serum TSH level as a significant variable associated with worse SAS (Beta = −0.695 and *p*=0.043) and SDS (Beta = −3.133 and *p* < 0.001) scores 3 months after surgery. FT4 was independently associated with SAS scores (Beta = −0.202 and *p* < 0.001). Patients with middle ATA risk had a significantly higher level of SDS scores (*p*=0.033). *Conclusion*: We confirmed that cancer-related anxiety and depression among DTC patients significantly alleviated 3 months after surgery. TSH suppression therapy has profound effects on cancer-related anxiety and depression, and the degree of anxiety and depression significantly deteriorated with the decrease of TSH level.

## 1. Introduction

Generally acknowledged as the most common endocrine-related malignant tumor, thyroid cancer (TC) encompasses a broad spectrum of pathological types including medullary thyroid carcinoma (MTC), papillary thyroid cancer (PTC), follicular thyroid cancer (FTC), and anaplastic thyroid cancer (ATC) [1]. Due to rapid lifestyle changes and widespread application of high resolution screening technologies, the prevalence of thyroid cancer has dramatically soared threefold over the past thirty years and made it way onto the most commonly diagnosed cancer among young women under 30 years old in China [2]. This substantial increase of incidence, primarily comprising PTC, seems to ascribe widespread diagnosis of subclinical disease [3]. The currently widely recognized clinical guidelines of differentiated thyroid cancer (DTC) is radical resection, followed by adjuvant radioactive iodine 131 therapy (RAI), a decade of thyroid-stimulating hormone (TSH) suppressive therapy, and life-time TSH replacement therapy [4].

Despite savage controversies center on the postsurgical management of DTC has been built up, TSH suppressive therapy, which aims to inhibit residual neoplastic tissue growth by exogenous levothyroxine, is widely recognized as the cornerstone of postoperative treatment [5]. However, emerging evidences indicate that subclinical hyperthyroidism (SCH) induced by TSH suppressive therapy could leave deleterious consequences on multiple organs and systems, for example, bone mineral density loss [6], cognitive function decline [7], as well as cardiac diastolic function impairment [8].

Steming from the fear about their impending mortality, uncertainty about social role reversal, as well as emotional distance between family members, cancer-related depression and anxiety, is a common but consistently underestimated predicament happened up to one third of patients with advanced cancer [9]. Moreover, without proper intervention, perennial psychological distress may in turn diminish the tolerability of chemoradiotherapy, treatment adherence, and social functioning, reinforcing a vicious cycle. For decades, the connection between hypothalamic pituitary thyroid (HPT) axis and neurological disorders has been gradually unraveled. It is confirmed that thyroid hormone fluctuation was associated with a wide variety of neuropsychiatric manifestations ranging from memory deficit, cognition impairment to tremor and acute psychoses [10]. For example, severe hypothyroidism, which commonly presented as depression, delirium, dementia, is also associated with decreased fine motor performance, reversible cognitive impairments, as well as memory decrement [11, 12]. On the other hand, aberrant elevation of serum TSH is a predictive biomarker of severe anxiety and suicide risk among patients suffering from major depressive disorder (MDD) [13, 14]. The TSH level was also a modifiable risk factor of depressive symptoms among general population with no clinical or laboratory evidence of thyroid dysfunction [15].

Exponential growth in incidence coupled with a 5-year relative survival rate reaching 98% [16], turn quality of life, instead of the traditional survival problem, a more alarming situation during the illness trajectory of DTC patients [17]. Nevertheless, previous study has demonstrated that favorable prognosis did not guarantee high health-related quality of life (HRQoL) [18]. On the contrary, the overall HRQoL of TC was basically consistent with that of cancers with poor prognosis such as glioma, colorectal cancer, even lower than that of breast cancer and cervical cancer [19–21]. Along with the alteration from traditional medical model to biopsychosocial modal, the therapeutic effect evaluation has also extended from clinical symptoms to social and psychological dimensions [22]. Therefore, it is of great concern to identify possible risk factors that are highly related to postoperation mood disorders and unsatisfying HRQoL.

This study first aimed to understand whether TSH suppressive therapy affect short-term postoperative cancer-related depression and anxiety among DTC patients. The second aim was to evaluate short-term postoperative psychological problems and whether they were associated with baseline parameters, fatigue, sleep quality, and illness perception. Our third aim was to investigate whether postoperative psychological problems could interfere with patients' quality of life.

## 2. Methods

### 2.1. Patients

This was a prospective, observational study. This study involved TC patients who consecutively admitted to the inpatient department of the hospital between 1^st^ June 2020 and 31^th^ February 2021. It was approved by Medical Ethics Committee of Shandong Provincial Hospital.

Inclusion criteria: initial diagnosis and no signs of distant metastasis. Exclusion criteria: patients with preexisting hypothyroidism, hyperthyroidism, neurological, or psychiatric disorders; age < 18; female patients who were pregnant; and patients with a familial history of mental health issues. Detailed surgical options (hemithyroidectomy or total thyroidectomy, with or without cervical lymph node dissection) were selected according to patients' condition. All participants were fully aware of their medical conditions. Patients requiring radioactive iodine therapy were fully informed the aim and necessity of RAI.

**Figure 1:**
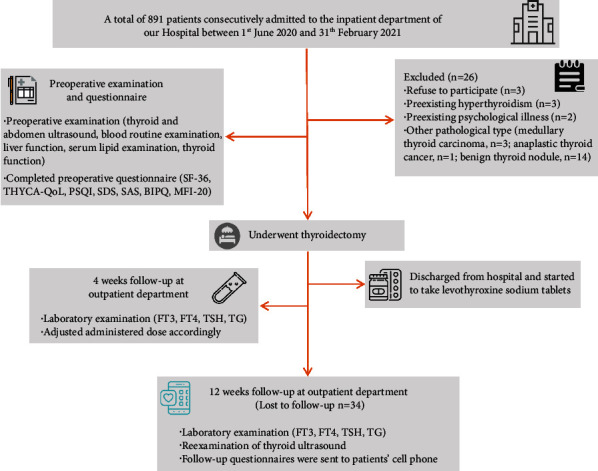
The flow diagram of this study's research method.

TSH suppressive therapy was applied after surgery. Thyroid function test (TG: thyroglobulin (only for patients underwent total thyroidectomy), FT3: free triiodothyronine, FT4: free thyroxine, TSH) were scheduled on postoperative week 4 and week 12 at the outpatient clinic. All patients were required to complete the questionnaires by themselves the day before surgery, and another one at the outpatient clinic when reexamined at three months after surgery. The flow diagram of this study's research method is shown in Figure 1.

### 2.2. Questionnaires

#### 2.2.1. 36-Item Short-Form Health Survey (SF-36)

SF-36, which consisted of 36 questions evaluating 8 domains (BP: bodily pain, GH: general health, MH: mental health, PF: physical functioning, RE: role emotional, RP: role physical, SF: social functioning, and VT: vitality), was applied to measure HRQoL among thyroid cancer patients [23–29].

#### 2.2.2. Thyroid Cancer-Specific Quality of Life (THYCA-QoL)

THYCA-QoL, which consisted of 24 items measuring seven symptom scales and six single items, was applied to evaluate thyroid cancer- or therapy-related side effects [30–32].

#### 2.2.3. Pittsburgh Sleep Quality Index (PSQI)

PSQI, which consisted of 19 items measuring seven dimensions, was used to evaluate sleep quality over the past months [33–37].

#### 2.2.4. Self-Rated Depression Scale (SDS) and Self-Rated Anxiety Scale (SAS)

The severity of anxiety and depression symptoms over the past week was assessed by the Zung's Chinese version of the SAS [38–42] and SDS [43–47]. SDS score ≥53 points and SAS score ≥50 points indicated existing depression and anxiety symptoms, respectively.

#### 2.2.5. Brief Illness Perception Questionnaire (BIPQ)

BIPQ [48–51], which consisted of 9 items covering 3 aspects: cognitive, emotional, and illness comprehensibility, was applied to evaluate illness perception.

#### 2.2.6. Multidimensional Fatigue Inventory (MFI-20)

MFI-20 was a 20-item self-reported questionnaire stipulated to reflect five dimensions (GF: general fatigue; MF: mental fatigue; RA: reduced activity; RM: reduced motivation; and PF: physical fatigue) [52–54].

### 2.3. Statistical Analysis

SPSS Statistics, R Studio, and Graphics were used for statistical analysis. Differences of psychosocial indicators were investigated using the Wilcoxon rank sum test. Bivariate correlation analysis between sociodemographic, clinical characteristics and SAS and SDS scores were accomplished using Pearson, Spearman, and Kendall correlations. Univariate and multivariate linear and logistic regression analysis were also performed. The association between different predictors and dependent variable over time was carried out by using fully adjusted linear mixed models. The fixed effect part of the linear mixed model explaining each score included age, education, children, sex, mean duration of chief complaint, modus operandi, histopathology, and multifocal tumor. *p* < 0.05 was considered statistically significant.

## 3. Results

### 3.1. Baseline Patient Characteristics

831 DTC patients were ultimately included in this study (Figure 1). Most of the patients were female (75.5%) in their mid 40s, finished nine-year compulsory education (69.7%), and physically fit without chronic conditions (75.8%). The mean time from first discovery to hospitalization was 9.26 months. 484 (58.2%) patients underwent fine needle aspiration cytology (FNAC), and the average time interval between FNAC and hospitalization was 0.92 months (Table 1).

Total thyroidectomy and unilateral lobectomy plus isthmectomy were two kinds of standard surgical options that were roughly equally applied (49% vs. 51%). A total of 630 (75%) patients had lymph node (LN) dissection in central neck (CN) region and the metastasis rate was 24.7 ± 35.2%, while 126 (15.2%) patients had LN dissection in lateral neck region and the metastasis rate was 25.3%. The most common pathological pattern was micro-PTC (less than 1 cm, 72%), followed by macro-PTC (larger than 1 cm, 26%), then FTC (2%), and 32.4% of DTC patients exhibited multifocal tumor.

### 3.2. Pre- and Postoperation Changes of Psychological State, Qol, BIPQ, and PSQI

**Figure 2:**
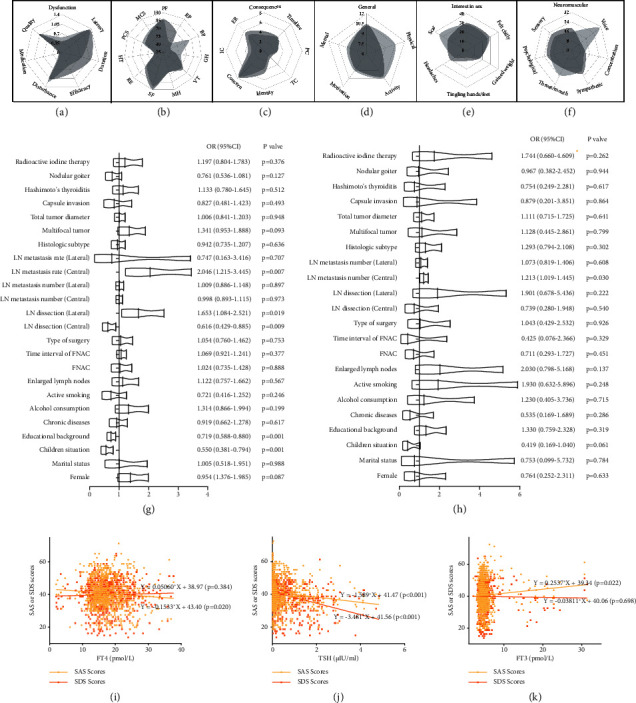
Radar graph displaying the score distribution of (a) PSQI, (b) SF-36, (c) BIPQ, (d) MFI-20, (e) THYCA-QoL single items, and (f) THYCA-QoL single scales. Univariate logistic regression models were created to evaluate the association of clinically significant predictor variables with short-term postoperative (g) anxiety and (h) depression. Single linear regression models were created to evaluate the association of (i) FT4, (j) TSH, and (k) FT3 with SAS (yellow) and SDS (orange) scores.

Compared with preoperative values, mean scores of SAS and SDS (Table 2 and Figures 2(a), 2(b), 2(c), 2(d), 2(e), and 2(f)) as well as the incidence of anxiety (41.5% vs. 22.1%) and depression (22.5% vs. 2.4%) significantly decreased 3 months after surgery.

Except for GF, deterioration in total MFI-20 scores as well as multiple subscales (PF, RM, and MF) were present 3 months after surgery (*p* < 0.05). The total scores of PSQI and two of its subscales, sleep duration and efficiency, decreased significantly over 3 months (*p* < 0.001), indicating improved sleep quality after operation. However, sleep disturbance and daytime dysfunction significantly increased (*p* < 0.001), and there was no evident difference in sleep quality, latency, and medication use. The mean score for illness perception, measured by BIPQ, was 47.95 before surgery, and 46.13 after surgery (*p* < 0.001), implying that DTC patients had a more positive change of illness perception. DTC survivors tended to have more confidence in “Consequences,” “Identity,” and “Emotional response” but less confidence in “Treatment control” and “Illness coherence” (*p* < 0.001).

As for THYCA-QoL scores, DTC survivors had more complaints about scar and voice 3 months after surgery (*p* < 0.001), whereas complaints about other thyroid-related symptoms such as felt chilly, tingling hands/feet, and headaches significantly ameliorated (*p* < 0.05). As for health condition measured by SF-36, both physical and mental wellbeing (*p* < 0.001) of DTC patients significantly meliorated after surgery. The scores for 5 domains (PF, RP, VT, SF, and RE; *p* < 0.05) decreased significantly from baseline level after treatment, meanwhile, BP and GH remarkably improved (all *p* < 0.001).

### 3.3. Linear Mixed-Effects Models

Linear-mixed model was performed to explore the existence of time-varying changes in SAS and SDS scores and discriminate the fixed effect of clinical variables relevant to psychological state changes (Table 3). For personal and clinical characteristics, low educational background (SAS, *β* = 1.392; SDS, *β* = 1.622; and *p* < 0.05), without children (SAS, *β* = 4.068; SDS, *β* = 1.873; and *p* < 0.01), FNAC (SAS, *β* = −0.981; SDS, *β* = −2.583; and *p* < 0.05), and multifocal tumor (SAS, *β* = −1.287; SDS, *β* = −2.681; and *p* < 0.05) were main effects for both anxiety and depression.

Compared with follicular thyroid carcinoma, patients suffering from papillary thyroid micro-carcinoma and papillary thyroid carcinoma were likely to have lower SAS scores (6.932 and 7.042 points, respectively, *p* < 0.001). The estimated coefficient of age (*β* = 0.055) and duration of the chief complaint (*β* = 0.030) were positive, meaning that over the whole observation time, SAS scores increased with age and time from presentation. Moreover, patients without plans to RAI tended to have lower SDS scores (*β* = −2.910, *p* < 0.001).

### 3.4. Factors Associated with Short-Term Postoperative Anxiety and Depression

#### 3.4.1. Demographics and Clinical Characteristics

Correlation analysis (Table 1) and univariate logistic regression analysis (Figure 2(g)) indicated that children situation, educational background, preoperative FNAC, LN dissection, and LN metastasis rate of CN region were closely associated with the presence of anxiety at postoperative 3 months. More specifically, highly educated patients (OR = 0.719), who had children (OR = 0.550) and underwent LN dissection of CN region (OR = 0.616) were less likely to suffer from short-term postoperative anxiety. On the contrary, patients underwent LN dissection of lateral neck region (OR = 1.653) were more likely to develop short-term postoperative anxiety. Multivariate logistic regression analysis also confirmed this verdict (Table 4).

On correlation analysis (Table 1), significant covariates included preoperative FNAC, LN dissection of lateral neck region, multifocal tumor, LN metastasis number/rate of the central and lateral neck region, and plans to RAI were proved to be strongly related to the presence of short-term postoperative depression (*p* < 0.05). Our research also demonstrated that, with the increasing of metastasis number of central neck region (*p*=0.030 and OR = 1.213) and metastasis rate of central (*p*=0.012 and OR = 5.115) and lateral neck region (*p*=0.007 and OR = 44.956), the likelihood of DTC survivors falling into despondency noticeably rose (Figure 2(h)). In addition, our findings corroborated that anxiety symptoms significantly deteriorated with the increase of the mean duration of the chief complaint (Beta = 0.041 and *p*=0.003) and levothyroxine dose (Beta = 0.133 and *p* < 0.001; Table 4).

According to Kendall's and Pearson's correlation test, anxiety and depression were irrelevant to age, sex, marital status, chronic diseases, concomitant Hashimoto thyroiditis or nodular goiter, bad addiction such as alcohol consumption, and active smoking. Anxiety and depression were also not associated with preoperative lymph nodes enlargement, modus operandi, histologic subtype, total tumor diameter, and capsule invasion.

#### 3.4.2. MFI-20, BIPQ, and PSQI

We also analyzed the relationship between fatigue, illness perception, sleep quality, and the severity of anxiety and depression. Correlation analysis and univariate linear regression analysis showed that higher level of total MFI-20, general fatigue, physical fatigue, reduced motivation, total BIPQ, consequences, identity, and emotional response scores were correlated with more serious anxiety. While higher total PSQI score was correlated with more impaired anxiety (Table 2). On the other hand, higher total MFI-20, general fatigue, reduced motivation, mental fatigue, sleep quality, sleep efficiency, and consequences were positively correlated with SDS score (Table 2). Multiple linear regression analysis further verified that consequences was the key risk factors for short-term postoperative anxiety, and total MFI-20 scores and mental fatigue were the key risk factors for short-term postoperative depression (Table 4).

#### 3.4.3. Thyroid Function

Correlation analysis and univariate linear regression analysis found that anxiety at postoperative 3 months was associated with declined FT4 and TSH, as well as elevated FT3 (Figures 2(i), 2(j), and 2(k)); furthermore, depression at postoperative 3 months was associated with declined TSH (Figure 2(j)). Multiple linear regression analysis also identified the serum TSH level as a significant variable associated with worse SAS (*p*=0.043) and SDS (*p* < 0.001) scores 3 months after surgery. FT4 was independently associated with SAS scores (Table 4). We then arbitrarily divided patients into 2 groups (low risk, *n* = 603 and middle risk, *n* = 228) according to the American Thyroid Association (ATA) risk criteria that define the risk of persistent/recurrent disease in DTC patients [55–58], and psychosocial indicators were compared using the *t*-test; the results are presented in Table 5. Middle-risk patients were found to have significantly higher level of SDS (39.62 ± 8.64 vs. 40.5 ± 7.86, *p*=0.033).

### 3.5. Short-Term Postoperative Anxiety, Depression, and QoL

Univariate linear regression analysis was applied to explore the impact of anxiety and depression on QoL (THYCA-QoL, SF-36). For THYCA-QoL (Table 2), anxiety was significantly associated with symptoms like chilly, tingling hands/feet, and headaches (*p* < 0.05). Depression was significantly associated with symptoms such as scar, felt chilly, headaches, neuromuscular, and voice (*p* < 0.05). For SF-36, higher SAS scores was significantly associated with lower GH (*p*=0.046), VT (*p*=0.005), MH (*p* < 0.001), SF (*p*=0.024), and mental component summary (*p*=0.001). Higher SDS scores were significantly associated with higher BP (*p*=0.029).

## 4. Discussion

Apart from physical and financial challenges, cancer-related depression and anxiety is another inevitable predicament which could increase patients' burden to live harder. First, we deliberated the longitudinal association between sociodemographic, clinical characteristics and psychological state. In consistent with Tae's work [59], we confirmed the definite alleviation of anxiety and depression among DTC patients 3 months after surgery. Anxiety (41.5%) and depression (22.5%) were highest at pretreatment and gradually decreased to 22.1% and 2.4%, respectively. As anticipated, sustained postoperative anxiety and depression were closely related to more aggressive tumor phenotype, higher grading of pathological histology (e.g., follicular thyroid carcinoma), and schedule of RAI in the near future. Aiming at measuring patients' mental health at different time points, the linear-mixed model was used to model variation within and between individuals, as well as changes between different time points. It seemed that fixed effects (including low educational background, no children, preoperative FNAC, and longer wait times for surgery) could continuously impact on anxiety and depression before and 3 months after the operation. In addition, in contrast to the traditional idea of women being more vulnerable to cancer-related psychological disorders [30], our research indicated that there was no gender difference in cancer-related anxiety and depression among DTC patients.

Second, we emphatically focused on the forecasting factors associated with short-term postoperative anxiety and depression, so as to discriminate high risk patients in advance. Our study for the first time demonstrated that lymph node dissection and metastasis were independent risk factors for short-term postoperative anxiety and depression in DTC patients. It is worth noting that lymph node dissection of central neck region was a protective factor for anxiety, and quite the contrary, lymph node dissection of lateral neck region was risk factor for anxiety.

Third, we elaborated the roles of fatigue, illness perception and sleep quality played in short-term postoperative psychological state changes. Haymart et al. have emphasized that substantial fatigue 2–4 years after surgery turned out to be one of the most common complaints among TC survivors [60]. Our study discovered a significant exacerbation in multiple subscales of MFI-20 (total MFI-20 scores, physical fatigue, reduced motivation, and mental fatigue) after operation. Single linear regression indicated that self-report fatigue among DTC survivors were significant risk factors of postoperative anxiety and depression. Furthermore, multiple linear regression also confirmed that total MFI-20 scores and mental fatigue were the key risk factors for short-term postoperative depression. We also found that the total score of PSQI, sleep duration, and sleep efficiency significantly decreased 3 months after surgery, implying an obviously melioration in sleep quality after operation. We also discovered that total PSQI scores were correlated with short-term postoperative anxiety, sleep quality, and efficiency were correlated with depression; however, multifactor linear regression did not achieve statistical significance. The BIPQ results revealed that DTC survivors generally viewed their condition as a relatively transitory process which still with many unknowns. They tended to adopt a considerable skeptical attitude toward treatment efficacy and so were more concerned about their disease. Besides, DTC patients had a more positive change of illness perception 3 months after surgery. It was clearly illustrated in our study that disease uncertainty, negative emotional response, as well as concern for the effects of illness on everyday life were positively correlated to short-term postoperative anxiety. Multifactor linear regression once again corroborated that concern for the effects of illness on everyday life was a crucial predictive factor of anxiety 3 months after surgery.

Another objective of our research was the effect of TSH suppressive therapy on short-term postoperative psychological state changes. Aiming to inhibit residual neoplastic tissue growth and prevent recurrence, TSH suppressive therapy has been widely recognized as the common treatment protocol for over half a century despite its dubious clinical effectiveness [61]. It is well established that iatrogenic subclinical hyperthyroidism, characterized by subnormal TSH level [62], was responsible for multiple undesired adverse effects including osteoporosis [63], atrial fibrillation, aortic stiffness [64], left ventricular mass enlargement [65], and cognitive function decline. On the other hand, HPT axis alterations as well as thyroid dysfunction have been universally accepted as mood modulators, which could serve as novel therapeutic targets for mood disorders [66, 67]. However, the exact role of thyroid hormonal levels in mood disorders has always been a controversial issue. Although it is complicated to compare our study to other cross-sectional analysis, it is possible that our findings could expand the results of other previous studies on subjects with thyroid cancer and psychological disorder. In the present study, we further reported that the short-term upheaval in the TSH level was negatively correlated with SAS and SDS scores, meaning that as the TSH level continued to fall, the degree of anxiety and depression significantly deteriorated. One possible explanation is that DTC patients tended to be female (75.5%, M/F ratio = 2.3 : 1) in their early 40s. Philibert et al. found that lower TSH level was associated with current depressive syndrome in young adults [68]. Lotufo et al. has reported that sex may play a substantial role in the relationship between depressive symptoms and TSH. The prevalence of depressive symptoms was approximately 1.92 times higher in men and 35% lower in women in the highest TSH tertile than the lowest TSH tertile [69]. On the other hand, the TSH level of DTC patients undergoing TSH suppression therapy is far below the normal range. Due to the effect of levothyroxine, the negative feedback of 5-hydroxytryptamine-thyrotropic hormone releasing hormone-TSH is forced to interrupt, creating a false “TSH blunting” state. Low TSH response has been repeatedly described in approximately 25% of the patients with major depression disorder [70]. In addition, it has been illuminated in a recently published article that DTC patients with suppressed TSH were inclined to have more prevalent depression, anxiety disorders, and sleep problems; furthermore, the TSH level was weakly but negatively correlated with anxiety sensitivity index and PSQI scores, while the duration of LT4 was positively correlated with beck depression inventory scores [71].

Last but not least, we deliberated the influence of anxiety and depression on quality of life. Comparing with preoperative values, in addition to “problems with scar,” DTC survivors had more complaints about “voice” and “throat/mouth” without definitive evidence of subsistent vocal cord paralysis, which probably due to operation induced dysphonia even in the absence of vocal cord immobility [72]. Anxiety was positively related to all of THYCA-QoL scales (Neuromuscular, Voice, Concentration, Sympathetic, Throat/mouth, Psychological, and Sensory), anxiety was also negatively related to MH, SF, and physical component summary in SF-36, indicating that it was a major source of poor quality of life. On the other hand, depression was significantly associated with all of THYCA-QoL scales except for “Sympathetic,” and “Throat/mouth.” Depression was positively related to BP. Previous studies have substantiated that TSH suppression therapy was one of the principal elements interrelated to unsatisfying quality of life [73]. Based on our research, we raised a hypothesis that postoperative anxiety and depression induced by long-term maintenance of low serum TSH level might be a significant mechanism of subpar quality of life.

This study had a few limitations. First, this was a single center study and the sample size was relatively small. Second, we did not set up a control group. Third, longer follow-up duration may be worthwhile to investigate time-dependent effects of TSH suppression therapy on cancer-related anxiety and depression.

All in all, we confirmed the definite alleviation of anxiety and depression among DTC patients 3 months after surgery. TSH suppression therapy has profound effects on cancer-related anxiety and depression, the degree of anxiety and depression significantly deteriorated with the decrease of the TSH level. We suggest that dynamic monitoring and early intervention of psychological states should be carried out among DTC patients, especially among patients with risk factors of developing cancer-related anxiety and depression.

## Figures and Tables

**Table 1:** Brief description and correlation analysis between anxiety, depression, and sociodemographic, clinical characteristics of DTC^1^ patients.

Characteristics	Values	Correlation	
		P1 (SAS^2^)	P2 (SDS^3^)
Mean age since diagnosis (years, mean ± SD)	44.25 ± 11.68	0.054	0.528
Female, *n* (%)	627 (75.5%)	0.875	0.272
Marital status, *n* (%)	—	0.424	0.506
With partner	777 (93.5%)	—	—
Without partner	54 (6.5%)	—	—
Children situation, *n* (%)	—	<0.001	0.470
0	185 (22.3%)	—	—
≥1	646 (77.7%)	—	—
Educational background, *n* (%)	—	0.027	0.374
≤nine-year compulsory education primary school	252 (30.3%)	—	—
High or technical school	270 (32.5%)	—	—
≥College graduate	309 (37.2%)	—	—
Chronic diseases^4^, *n* (%)	—	0.958	0.743
0	630 (75.8%)	—	—
1	176 (21.2%)	—	—
≥2	25 (3.0%)	—	—
Alcohol consumption, *n* (%)	141 (17.0%)	0.078	0.289
Active smoking, *n* (%)	97 (11.7%)	0.192	0.078
Duration of the chief complaint^5^ (months, mean ± SD)	9.26 ± 18.40	0.001	0.191
Enlarged lymph nodes^6^, *n* (%)	177 (21.3%)	0.257	0.136
FNAC^7^, *n* (%)	484 (58.2%)	0.211	0.002
Affirmative cancer cells	302 (36.3%)	—	—
Suspicious cancer cells	19 (2.3%)	—	—
No trace of cancer cells	163 (19.6%)	—	—
Time interval of FNAC^8^ (months, mean ± SD)	0.92 ± 1.30	0.018	0.903
Type of surgery, *n* (%)	—	0.942	0.819
Unilateral lobectomy plus isthmectomy	407 (49%)	—	—
Total thyroidectomy	424 (51%)	—	—
Number of metastasis lymph nodes (*n*, mean ± SD)	—	—	—
Central neck region	0.87 ± 1.64	0.543	0.046
Lateral neck region	2.74 ± 2.94	0.365	0.011
Lymph node metastasis rate^9^ (%, mean ± SD)	—		
Central neck region (630, 75%)	24.7 ± 35.2%	0.089	0.026
Lateral neck region (126, 15%)	25.3 ± 25.7%	0.268	0.035
Histologic subtype, *n* (%)	—	0.547	0.871
Papillary thyroid microcarcinoma	598 (72%)	—	—
Papillary thyroid carcinoma	216 (26%)	—	—
Follicular thyroid carcinoma	17 (2%)	—	—
Multifocal tumor, *n* (%)	269 (32.4%)	0.195	0.006
Total tumor diameter^10^ (cm, mean ± SD)	1.09 ± 0.91	0.767	0.525
Capsule invasion, *n* (%)	93 (11.2%)	0.668	0.235
Hashimoto's thyroiditis, *n* (%)	206 (24.8%)	0.553	0.211
Nodular goiter, *n* (%)	297 (35.7%)	0.717	0.205
Plans to radioactive iodine therapy, *n* (%)	166 (20%)	0.677	0.037
Levothyroxine dose^11^ (*μ*g, mean ± SD)	110.26 ± 15.28	<0.001	<0.001

^1^DTC: differentiated thyroid cancer; ^2^SAS: self-rated anxiety scale; ^3^SDS: self-rated depression scale; ^4^chronic diseases: number of chronic diseases including hypertension, diabetes, coronary heart diseases, and renal failure; ^5^duration of the chief complaint: the time from first discovery to hospitalization; ^6^enlarged lymph nodes: preoperative ultrasound showed lymph nodes enlargement in lateral neck region; ^7^FNAC: fine needle aspiration cytology; ^8^time interval of FNAC: time interval between FNAC and operation; ^9^lymph node metastasis rate: the ratio between metastatic and examined lymph nodes; ^10^total tumor diameter: the total tumor diameter among multifocal tumor was the sum of diameters of each malignant nodule; ^11^levothyroxine dose: levothyroxine dose at 3 months after surgery. P1 and P2 represent the *p* valve of correlation analysis between sociodemographic, clinical characteristics and SAS and SDS scores.

**Table 2:** Pre- and postoperation of psychosocial indicators and its relationship with anxiety and depression.

Characteristics	Values		P1	Correlation		Single linear regression	
				SAS	SDS	SAS	SDS
	Preoperative	12 weeks		P2	P3	Beta1^4^	Beta2^5^
Anxiety	49.04 ± 7.45	40.69 ± 9.54	<0.001	—	—	—	—
Depression	44.61 ± 10.10	39.86 ± 8.43	<0.001	—	—	—	—
MFI-20	46.42 ± 7.41	49.29 ± 11.62	<0.001	0.005	0.010	**0.080**	**0.066**
General fatigue	10.40 ± 2.46	10.17 ± 3.11	0.026	0.011	0.014	**0.270**	**0.233**
Physical fatigue	8.34 ± 2.63	10.53 ± 2.91	<0.001	0.001	0.094	**0.369**	0.170
Reduced activity	10.14 ± 2.66	10.38 ± 2.65	0.058	0.380	0.601	0.110	0.059
Reduced motivation	8.74 ± 2.41	9.08 ± 2.57	0.005	0.007	0.017	**0.351**	**0.274**
Mental fatigue	8.70 ± 2.65	9.13 ± 3.17	0.016	0.074	0.002	0.188	**0.295**
THYCA-QoL single items							
Problems with scar	23.71 ± 30.89	34.22 ± 34.81	<0.001	0.687	0.079	0.004	0.015
Felt chilly	28.80 ± 30.60	26.07 ± 30.38	0.031	0.340	0.965	−0.010	0.000
Tingling hands/feet	11.39 ± 20.79	7.70 ± 17.38	<0.001	0.001	0.003	**0.065**	**0.050**
Gained weight	16.65 ± 24.10	17.05 ± 24.42	0.803	0.885	0.869	0.002	−0.002
Headaches	20.50 ± 22.93	15.56 ± 21.30	<0.001	0.029	0.151	**−0.034**	0.020
Less interest in sex	29.60 ± 25.68	28.16 ± 26.81	0.114	0.076	0.015	**−0.022**	**−0.026**
THYCA-QoL scales							
Neuromuscular	18.65 ± 15.00	15.50 ± 15.28	<0.001	0.001	<0.001	**0.128**	**0.087**
Voice	10.23 ± 16.92	30.08 ± 26.37	<0.001	0.269	0.227	0.014	0.013
Concentration	12.47 ± 18.37	11.25 ± 18.55	0.078	0.549	0.002	0.011	**0.049**
Sympathetic	21.72 ± 19.34	19.21 ± 20.34	<0.001	0.936	0.885	−0.001	0.002
Throat/mouth	16.82 ± 14.42	23.25 ± 17.60	<0.001	0.680	0.302	−0.008	0.017
Psychological	21.35 ± 16.50	18.71 ± 17.92	<0.001	0.669	0.005	−0.008	**0.046**
Sensory	21.50 ± 19.49	18.17 ± 18.94	<0.001	0.086	0.387	−0.030	0.013
Total PSQI	5.99 ± 2.73	5.52 ± 2.97	<0.001	0.004	0.069	**−0.321**	−0.179
Sleep quality	1.09 ± 0.65	1.07 ± 0.62	0.355	0.240	0.446	−0.627	−0.359
Sleep latency	1.34 ± 0.94	1.29 ± 0.89	0.363	0.005	0.005	**−1.037**	**−0.920**
Sleep duration	1.11 ± 0.83	0.74 ± 0.83	<0.001	0.181	0.313	−0.534	−0.356
Sleep efficiency	0.81 ± 1.06	0.54 ± 0.86	<0.001	0.095	0.963	−0.643	−0.016
Sleep disturbance	1.08 ± 0.49	1.20 ± 0.53	<0.001	0.011	0.003	**−1.588**	**−1.614**
Sleep medication use	0.14 ± 0.54	0.10 ± 0.44	0.088	0.063	0.257	−1.396	0.753
Daytime dysfunction	0.43 ± 0.67	0.57 ± 0.69	<0.001	0.342	0.451	−0.457	−0.321
Total BIPQ	47.95 ± 11.84	46.13 ± 10.07	<0.001	<0.001	0.087	**0.137**	0.050
Consequences	4.17 ± 2.88	3.35 ± 2.49	<0.001	<0.001	0.005	**1.037**	**0.330**
Timeline	4.10 ± 3.19	3.88 ± 3.19	0.388	0.148	0.454	0.150	0.069
Personal control	7.20 ± 2.64	7.22 ± 2.69	0.784	0.061	0.347	−0.231	−0.102
Treatment control	8.14 ± 2.38	8.61 ± 2.00	<0.001	0.208	0.665	−0.210	0.064
Identity	4.59 ± 3.10	3.57 ± 2.60	<0.001	0.001	0.289	**0.408**	0.119
Concern	7.68 ± 2.77	7.53 ± 2.85	0.582	0.065	0.378	0.214	0.091
Illness coherence	6.57 ± 2.48	7.28 ± 2.36	<0.001	0.300	0.129	0.146	0.188
Emotional response	5.51 ± 2.95	4.70 ± 2.93	<0.001	0.001	0.818	**0.367**	0.023
SF-36							
Physical functioning	88.24 ± 14.32	85.87 ± 13.76	<0.001	0.443	0.354	−0.018	0.020
Role physical	71.30 ± 32.39	47.59 ± 41.02	<0.001	0.327	0.625	−0.008	0.003
Bodily pain	40.71 ± 6.18	79.44 ± 14.66	<0.001	0.181	0.029	−0.030	**0.044**
General health	50.97 ± 20.32	60.37 ± 20.08	<0.001	0.046	0.712	**−0.033**	−0.005
Vitality	75.40 ± 15.37	71.25 ± 18.30	<0.001	0.005	0.991	**−0.051**	0.000
Mental health	65.39 ± 16.11	65.11 ± 18.18	0.670	<0.001	0.105	**−0.103**	−0.260
Social functioning	99.14 ± 21.16	93.68 ± 24.42	<0.001	0.024	0.170	**−0.031**	−0.160
Role emotional	71.68 ± 33.67	66.60 ± 40.06	0.040	0.233	0.852	−0.010	−0.001
HT^1^	42.87 ± 20.85	43.59 ± 25.04	0.972	0.943	0.203	−0.001	0.015
PCS^2^	62.81 ± 11.18	68.32 ± 16.62	<0.001	0.097	0.388	−0.033	0.015
MCS^3^	70.90 ± 15.07	74.02 ± 20.00	<0.001	0.001	0.380	−0.053	−0.013

	4 weeks	12 weeks	P1	P2	P3	Beta1^4^	Beta2^5^

TSH^6^	1.02 ± 1.48	0.49 ± 0.77	<0.001	<0.001	<0.001	**−1.589**	**−3.481**
FT3^7^	4.98 ± 1.53	5.32 ± 3.00	<0.001	0.022	0.698	**0.254**	−0.038
FT4^8^	18.83 ± 7.16	17.62 ± 5.04	0.010	0.020	0.384	**−0.153**	0.051
TG^9^	1.43 ± 2.22	1.50 ± 2.61	0.202	0.286	0.115	0.182	−0.248

^1^HT: reported health transition; ^2^PCS: physical component summary; ^3^MCS: mental component summary; ^4-5^: single linear regression model were created to evaluate the association of postoperative MFI-20, BIPQ, PSQI, THYCA-QoL, SF-36 with anxiety (SAS) and depression (SDS), bold indicates *p* < 0.05; ^6^TSH: reference range 0.35–4.94 *μ*IU/mL; ^7^FT3: reference range 2.43–6.01 pmol/L; ^8^FT4: reference range 9.01–19.05 pmol/L; ^9^TG: reference range 3.5–77 ng/ml.

**Table 3:** Linear mixed-effect model estimates of effects for thyroid hormones (FT3, FT4, and TSH), fatigue, sleep quality, and illness perception on anxiety and depression.

	Anxiety^a^ (SAS scores)					Depression^a^ (SDS scores)				*p*
	Estimate	SE^1^	95% CI^2^		*p*	Estimate	SE	95% CI		
			Inf^3^	Sup^4^				Inf	Sup	
Educational background (ref: college graduate)										
≤nine-year compulsory education	1.392	0.587	0.240	2.544	0.018	1.622	0.656	0.334	2.909	0.014
High or technical school	0.442	0.519	−0.575	1.460	0.394	0.492	0.580	−0.645	1.629	0.396
Female (ref: Male)	0.526	0.479	−0.414	1.467	0.272	−0.382	0.535	−1.432	0.668	0.476
Married (ref: Without partner)	0.398	0.943	−1.453	2.248	0.673	0.774	1.054	−1.293	2.841	0.463
Without children (ref: with children)	4.068	0.538	3.013	5.123	<0.001	1.873	0.601	0.694	3.052	0.002
Without family history of TC (ref: with)	3.284	1.593	0.159	6.409	0.039	−2.223	1.780	−5.714	1.269	0.212
Without chronic diseases (ref: with chronic diseases)	−0.763	0.494	−1.732	0.206	0.123	0.211	0.552	−0.872	1.293	0.703
Nonsmoker (ref: smoker)	−0.322	0.641	−1.579	0.935	0.615	1.130	0.716	−0.274	2.534	0.115
Nondrinker (ref: drinker)	−1.734	0.537	−2.788	−0.681	0.001	−0.496	0.600	−1.673	0.681	0.408
Without FNAC (ref: FNAC)	−0.981	0.407	−1.779	−0.182	0.016	−2.583	0.455	−3.475	−1.691	<0.001
Type of surgery (ref: unilateral lobectomy plus isthmectomy)										
Total thyroidectomy	0.172	0.524	−0.856	1.200	0.743	−1.882	0.586	−3.031	−0.733	0.001
Single tumor (ref: multifocal)	−1.287	0.523	−2.314	−0.261	0.014	−2.681	0.585	−3.828	−1.534	<0.001
Histologic subtype (ref: follicular thyroid carcinoma)										
Papillary thyroid micro-carcinoma	−6.932	1.571	−10.015	−3.850	<0.001	−3.340	1.756	−6.784	0.103	0.057
Papillary thyroid carcinoma	−7.042	1.516	−10.016	−4.067	<0.001	−3.202	1.694	−6.526	0.121	0.059
Without plans to radioactive iodine therapy (ref: with plans)	0.713	0.605	−0.474	1.899	0.239	−2.910	0.676	−4.236	−1.585	<0.001
Duration of the chief complaint	0.030	0.011	0.009	0.052	0.005	−0.007	0.012	−0.031	0.017	0.546
Total tumor diameter	−0.548	0.304	−1.145	0.049	0.072	−0.227	0.340	−0.894	0.440	0.504
Age	0.055	0.023	0.010	0.099	0.017	−0.026	0.026	−0.076	0.024	0.301
Time	8.326	0.406	7.529	9.123	<0.001	4.766	0.447	3.890	5.643	<0.001

^1^SE: standard error; ^a^: measured twice with a time interval of 3 months (preoperation and postoperation 3 months); ^b^: redundancy; ^2^CI: confidence interval; ^3^Inf: infimum; ^4^Sup: supremum.

**Table 4:** Multivariate linear and logistic regression model of risk factors associated with short-term postoperative anxiety and depression.

Characteristics	Multivariate logistic regression (SAS)				Multivariate logistic regression (SDS)			
	P1	OR^1^	Inf	Sup	P2	OR	Inf	Sup
Children situation	<0.001	0.512	0.351	0.746	0.124	0.134	0.01	1.733
Educational background	0.001	0.706	0.574	0.869		—		
LN^2^ dissection (central)	0.004	0.581	0.400	0.844		—		
LN dissection (lateral)	0.018	1.692	1.096	2.612		—		
Number of LN metastasis (central)	—				0.624	0.837	0.41	1.707
LN metastasis rate (central)	—				0.320	7.862	0.135	459.433
LN metastasis rate (lateral)	—				0.583	3.666	0.035	380.886

Characteristics	Multiple linear regression (SAS)				Multiple linear regression (SDS)			
	Unstandardized beta		P3		Unstandardized beta		P4	

Duration of the chief complaint	0.038		0.008		−0.020		0.169	
Levothyroxine dose	−0.343		<0.001		−0.140		<0.001	
TSH	−0.695		0.043		−3.133		<0.001	
FT3	0.145		0.102		—			
FT4	−0.202		<0.001		—			
MFI-20	−0.112		0.170		−0.166		0.036	
General fatigue	0.062		0.717		0.205		0.215	
Physical fatigue	0.243		0.139		—			
Reduced motivation	0.306		0.094		0.150		0.403	
Mental fatigue	—				0.491		0.005	
Total PSQI	−0.148		0.089		−0.095		0.290	
Sleep quality	—				0.037		0.067	
Sleep efficiency	—				0.031		0.138	
Total BIPQ	0.009		0.819		0.029		0.382	
Consequences	0.697		<0.001		0.150		0.267	
Identity	0.169		0.153		—			
Emotional response	−0.182		0.134		—			

^1^OR: odds ratio; ^2^LN: lymph node.

**Table 5:** Comparison of psychosocial indicators between groups (means ± SD).

Variables	Low risk^4^	Middle risk^5^	*F* value	*p* value
*N*	603	228	—	—
Anxiety	40.74 ± 9.39	40.58 ± 9.97	2.009	0.157
Depression	39.62 ± 8.64	40.5 ± 7.86	4.556	0.033
MFI-20	49.14 ± 11.74	49.2 ± 10.99	0.675	0.412
THYCA-QoL single items				
Problems with scar	34.49 ± 34.86	33.48 ± 34.77	0.021	0.886
Felt chilly	26.26 ± 30.1	25.58 ± 31.18	0.419	0.518
Tingling hands/feet	8.18 ± 17.9	6.43 ± 15.88	5.244	0.022
Gained weight	17.19 ± 25	16.67 ± 22.89	1.548	0.214
Headaches	15.81 ± 21.15	14.91 ± 21.72	0.013	0.908
Less interest in sex	28.08 ± 25.63	28.36 ± 29.76	8.19	0.004
THYCA-QoL scales				
Neuromuscular	15.7 ± 15.14	14.96 ± 15.67	0.094	0.759
Voice	30.32 ± 26.27	29.46 ± 26.7	0.067	0.796
Concentration	12.24 ± 19.3	8.63 ± 16.14	7.715	0.006
Sympathetic	19.54 ± 20.76	18.35 ± 19.18	1.727	0.189
Throat/mouth	23.31 ± 17.58	23.1 ± 17.68	0.001	0.974
Psychological	19.85 ± 18.34	15.72 ± 16.45	3.282	0.07
Sensory	18.77 ± 19.36	16.59 ± 17.73	2.734	0.099
Total PSQI	5.54 ± 2.97	5.44 ± 2.99	0.258	0.611
Total BIPQ	46.2 ± 10.11	45.96 ± 9.98	0.211	0.646
SF-36				
Physical functioning	85.55 ± 14.15	86.73 ± 12.66	0.997	0.318
Role physical	46.72 ± 41.04	49.89 ± 40.95	0.014	0.906
Bodily pain	79.11 ± 14.95	80.3 ± 13.86	0.34	0.56
General health	59.98 ± 20.11	61.43 ± 20	0.088	0.767
Vitality	70.95 ± 18.95	72.02 ± 16.48	2.936	0.087
Mental health	64.68 ± 18.54	66.25 ± 17.15	1.335	0.248
Social functioning	92.93 ± 24.95	95.67 ± 22.9	3.74	0.053
Role emotional	65.01 ± 40.13	68.86 ± 39.82	0.228	0.633
HT^1^	42.95 ± 25.58	45.29 ± 23.52	4.611	0.032
PCS^2^	67.84 ± 16.98	69.59 ± 15.58	1.367	0.243
MCS^3^	73.39 ± 20.76	75.7 ± 17.79	9.285	0.002

^1^HT: reported health transition; ^2^PCS: physical component summary; ^3^MCS: mental component summary; ^4-5^low risk and middle risk were according to the American Thyroid Association (ATA) risk criteria.

## Data Availability

The data that support the findings of this study are available from the corresponding author upon reasonable request.

## References

[1] Siegel R. L., Miller K. D., Jemal A. (2020). Cancer statistics, 2020. *CA: A Cancer Journal for Clinicians*.

[2] Chen W., Zheng R., Baade P. D. (2016). Cancer statistics in China, 2015. *CA: A Cancer Journal for Clinicians*.

[3] Morris L. G., Tuttle R. M., Davies L. (2016). Changing trends in the incidence of thyroid cancer in the United States. *JAMA Otolaryngol Head Neck Surg*.

[4] Basım P., Argun D., Özdenkaya Y. (2021). Self-reported medication adherence in differentiated thyroid cancer survivors: role of illness perception and medication beliefs. *Head & Neck*.

[5] Grani G., Ramundo V., Verrienti A., Sponziello M., Durante C. (2019). Thyroid hormone therapy in differentiated thyroid cancer. *Endocrine*.

[6] Moon J. H., Kim K. M., Oh T. J. (2017). The effect of TSH suppression on vertebral trabecular bone scores in patients with differentiated thyroid carcinoma. *Journal of Clinical Endocrinology and Metabolism*.

[7] Moon J. H., Ahn S., Seo J. (2014). The effect of long-term thyroid-stimulating hormone suppressive therapy on the cognitive function of elderly patients with differentiated thyroid carcinoma. *Journal of Clinical Endocrinology and Metabolism*.

[8] Pajamäki N., Metso S., Hakala T. (2018). Long-term cardiovascular morbidity and mortality in patients treated for differentiated thyroid cancer. *Clinical Endocrinology*.

[9] Tang S. T., Chen J. S., Chou W. C. (2016). Longitudinal analysis of severe anxiety symptoms in the last year of life among patients with advanced cancer: relationships with proximity to death, burden, and social support. *Journal of the National Comprehensive Cancer Network*.

[10] Larisch R., Kley K., Nikolaus S. (2004). Depression and anxiety in different thyroid function states. *Hormone and Metabolic Research*.

[11] Burmeister L. A., Ganguli M., Dodge H. H., Toczek T., DeKosky S. T., Nebes R. D. (2001). Hypothyroidism and cognition: preliminary evidence for a specific defect in memory. *Thyroid*.

[12] Smith C. D., Grondin R., LeMaster W., Martin B., Gold B. T., Ain K. B. (2015). Reversible cognitive, motor, and driving impairments in severe hypothyroidism. *Thyroid*.

[13] Shen Y., Wu F., Zhou Y. (2019). Association of thyroid dysfunction with suicide attempts in first-episode and drug naïve patients with major depressive disorder. *Journal of Affective Disorders*.

[14] Lang X., Hou X., Shangguan F., Zhang X. Y. (2020). Prevalence and clinical correlates of subclinical hypothyroidism in first-episode drug-naive patients with major depressive disorder in a large sample of Chinese. *Journal of Affective Disorders*.

[15] Kim E. Y., Kim S. H., Rhee S. J. (2015). Relationship between thyroid-stimulating hormone levels and risk of depression among the general population with normal free T4 levels. *Psychoneuroendocrinology*.

[16] McLeod D. S. A., Zhang L., Durante C., Cooper D. S. (2019). Contemporary debates in adult papillary thyroid cancer management. *Endocrine Reviews*.

[17] Kelly A., Barres B., Kwiatkowski F. (2019). Age, thyroglobulin levels and ATA risk stratification predict 10-year survival rate of differentiated thyroid cancer patients. *PLoS One*.

[18] Roerink S. H., de Ridder M., Prins J. (2013). High level of distress in long-term survivors of thyroid carcinoma: results of rapid screening using the distress thermometer. *Acta Oncologica*.

[19] Applewhite M. K., James B. C., Kaplan S. P. (2016). Quality of life in thyroid cancer is similar to that of other cancers with worse survival. *World Journal of Surgery*.

[20] Choi H. G., Park B., Ji Y. B., Tae K., Song C. M. (2019). Depressive disorder in thyroid cancer patients after thyroidectomy: a longitudinal follow-up study using a national cohort. *Otolaryngology-Head and Neck Surgery*.

[21] Goswami S., Mongelli M., Peipert B. J., Helenowski I., Yount S. E., Sturgeon C. (2018). Benchmarking health-related quality of life in thyroid cancer versus other cancers and United States normative data. *Surgery*.

[22] Li J., Zhang B., Bai Y., Liu Y., Zhang B., Jin J. (2020). Health-related quality of life analysis in differentiated thyroid carcinoma patients after thyroidectomy. *Scientific Reports*.

[23] Zhang C., Cai Y., Xue Y. (2021). Exploring the influencing factors of quality of life among the empty nesters in Shanxi, China: a structural equation model. *Health and Quality of Life Outcomes*.

[24] Wan C. F., Song T. (2022). Comparison of two different pulsed radiofrequency modes for prevention of postherpetic neuralgia in elderly patients with acute/subacute trigeminal herpes zoster. *Neuromodulation: Technology at the Neural Interface*.

[25] Liu Y. T., Lai J. Z., Zhai F. F. (2021). Right ventricular systolic function is associated with health-related quality of life: a cross-sectional study in community-dwelling populations. *Annals of Translational Medicine*.

[26] Li M. Y., Yang Y. L., Liu L., Wang L. (2016). Effects of social support, hope and resilience on quality of life among Chinese bladder cancer patients: a cross-sectional study. *Health and Quality of Life Outcomes*.

[27] Cao J., Zheng X., Wei W. (2021). Three-month outcomes of recovered COVID-19 patients: prospective observational study. *Therapeutic Advances in Respiratory Disease*.

[28] Li L., Wang H., Shen Y. (2002). Development and psychometric tests of a Chinese version of the SF-36 health survey scales. *Zhonghua Yufang Yixue Zazhi*.

[29] Lan Y., Cao L., Song Q. (2021). The quality of life in papillary thyroid microcarcinoma patients undergoing lobectomy or total thyroidectomy: a cross-sectional study. *Cancer Medicine*.

[30] Goldfarb M., Casillas J. (2016). Thyroid cancer-specific quality of life and health-related quality of life in young adult thyroid cancer survivors. *Thyroid*.

[31] Ahn J., Jeon M. J., Song E. (2020). Quality of life in patients with papillary thyroid microcarcinoma according to treatment: total thyroidectomy with or without radioactive iodine ablation. *Endocrinol Metab (Seoul)*.

[32] Lan Y., Luo Y., Zhang M. (2020). Quality of life in papillary thyroid microcarcinoma patients undergoing radiofrequency ablation or surgery: a comparative study. *Frontiers in Endocrinology*.

[33] Tsai P. S., Wang S. Y., Wang M. Y. (2005). Psychometric evaluation of the Chinese version of the pittsburgh sleep quality index (CPSQI) in primary insomnia and control subjects. *Quality of Life Research*.

[34] Yang F., Zhang Y., Qiu R., Tao N. (2021). Association of sleep duration and sleep quality with hypertension in oil workers in Xinjiang. *PeerJ*.

[35] Liu B., Song L., Zhang L. (2019). Sleep patterns and the risk of adverse birth outcomes among Chinese women. *International Journal of Gynecology and Obstetrics*.

[36] Zhang H., Zhao X., Li Y. (2020). Night sleep duration and sleep initiation time with hypertension in Chinese rural population: the Henan Rural Cohort. *The European Journal of Public Health*.

[37] Huang X., Li H., Meyers K. (2017). Burden of sleep disturbances and associated risk factors: a cross-sectional survey among HIV-infected persons on antiretroviral therapy across China. *Scientific Reports*.

[38] Shi X., Wang S., Wang Z., Fan F. (2021). The resilience scale: factorial structure, reliability, validity, and parenting-related factors among disaster-exposed adolescents. *BMC Psychiatry*.

[39] Wang Y., Meng Z., Pei J. (2021). Anxiety and depression are risk factors for recurrent pregnancy loss: a nested case-control study. *Health and Quality of Life Outcomes*.

[40] Luo Q., Zhou H., Yang R. (2021). Effect of care bundles on postoperative pain, negative emotions, and self-care ability of patients with acute dacryocystitis. *American Journal of Translational Research*.

[41] Pan Z., Huang Q., Jiang L. (2021). Application effects of targeted nursing model in patients undergoing thyroid surgery and its influence on patients’ negative emotions. *American Journal of Translational Research*.

[42] Qian H., Zhou J., Huang T. (2021). Comfort nursing can alleviate pain and negative emotion of patients after surgery for LVCFs and improve their living ability. *American Journal of Translational Research*.

[43] Zou P., Wang X., Sun L. (2018). Semen quality in Chinese college students: associations with depression and physical activity in a cross-sectional study. *Psychosomatic Medicine*.

[44] Zhang Y., An H., Xu L., Tao N. (2020). Relationship between depression, the family environment, and the coping styles of military recruits: a cross-section study. *Medicine (Baltimore)*.

[45] Liu Y., Li H., Xu X. (2020). The relationship between insecure attachment to depression: mediating role of sleep and cognitive reappraisal. *Neural Plasticity*.

[46] Zung W. W. (1967). Factors influencing the self-rating depression scale. *Archives of General Psychiatry*.

[47] Dugan W., McDonald M. V., Passik S. D., Rosenfeld B. D., Theobald D., Edgerton S. (1998). Use of the Zung Self-Rating Depression Scale in cancer patients: feasibility as a screening tool. *Psycho-Oncology*.

[48] Broadbent E., Petrie K. J., Main J., Weinman J. (2006). The brief illness perception questionnaire. *Journal of Psychosomatic Research*.

[49] Zhang N., Fielding R., Soong I. (2017). Psychometric assessment of the Chinese version of the brief illness perception questionnaire in breast cancer survivors. *PLoS One*.

[50] An J., Zhou H., Yang T. (2021). Relationship of psychological factors with daily activities and quality of life in patients with chronic obstructive pulmonary disease in a Chinese rural population. *Annals of Palliative Medicine*.

[51] Fan S. Y., Eiser C., Ho M. C., Lin C. Y. (2013). Health-related quality of life in patients with hepatocellular carcinoma: the mediation effects of illness perceptions and coping. *Psycho-Oncology*.

[52] Norup A., Svendsen S. W., Doser K. (2019). Prevalence and severity of fatigue in adolescents and young adults with acquired brain injury: a nationwide study. *Neuropsychological Rehabilitation*.

[53] Wintermann G. B., Weidner K., Strauss B., Rosendahl J. (2020). Single assessment of delirium severity during postacute intensive care of chronically critically ill patients and its associated factors: post hoc analysis of a prospective cohort study in Germany. *BMJ Open*.

[54] Pien L. C., Chu H., Chen W. C. (2011). Reliability and validity of a Chinese version of the multidimensional fatigue symptom inventory-short form (MFSI-SF-C). *Journal of Clinical Nursing*.

[55] Haugen B. R., Alexander E. K., Bible K. C. (2016). 2015 American thyroid association management guidelines for adult patients with thyroid nodules and differentiated thyroid cancer: the American thyroid association guidelines task force on thyroid nodules and differentiated thyroid cancer. *Thyroid*.

[56] Francis G. L., Waguespack S. G., Bauer A. J. (2015). Management guidelines for children with thyroid nodules and differentiated thyroid cancer: the American thyroid association guidelines task force on pediatric thyroid cancer. *Thyroid*.

[57] Haugen B. R. (2017). American thyroid association management guidelines for adult patients with thyroid nodules and differentiated thyroid cancer: what is new and what has changed?. *Cancer*.

[58] Ruben R., Pavithran P. V., Menon V. U., Nair V., Kumar H. (2019). Performance of ATA risk stratification systems, response to therapy, and outcome in an Indian cohort of differentiated thyroid carcinoma patients: a retrospective study. *European Thyroid Journal*.

[59] Song C. M., Bang H. S., Kim H. G., Park H. J., Tae K. (2021). Health-related quality of life after transoral robotic thyroidectomy in papillary thyroid carcinoma. *Surgery*.

[60] Hughes D. T., Reyes-Gastelum D., Kovatch K. J., Hamilton A. S., Ward K. C., Haymart M. R. (2020). Energy level and fatigue after surgery for thyroid cancer: a population-based study of patient-reported outcomes. *Surgery*.

[61] Parker W. A., Edafe O., Balasubramanian S. P. (2017). Long-term treatment-related morbidity in differentiated thyroid cancer: a systematic review of the literature. *Pragmatic and Observational Research*.

[62] McLeod D. S., Sawka A. M., Cooper D. S. (2013). Controversies in primary treatment of low-risk papillary thyroid cancer. *The Lancet*.

[63] Moon J. H., Jung K. Y., Kim K. M. (2016). The effect of thyroid stimulating hormone suppressive therapy on bone geometry in the hip area of patients with differentiated thyroid carcinoma. *Bone*.

[64] Gazdag A., Nagy E. V., Erdei A. (2015). Aortic stiffness and left ventricular function in patients with differentiated thyroid cancer. *Journal of Endocrinological Investigation*.

[65] Shargorodsky M., Serov S., Gavish D., Leibovitz E., Harpaz D., Zimlichman R. (2006). Long-term thyrotropin-suppressive therapy with levothyroxine impairs small and large artery elasticity and increases left ventricular mass in patients with thyroid carcinoma. *Thyroid*.

[66] Fischer S., Ehlert U. (2018). Hypothalamic-pituitary-thyroid (HPT) axis functioning in anxiety disorders. A systematic review. *Depression and Anxiety*.

[67] Min W., Liu C., Yang Y. (2012). Alterations in hypothalamic-pituitary-adrenal/thyroid (HPA/HPT) axes correlated with the clinical manifestations of depression. *Progress in Neuro-Psychopharmacology and Biological Psychiatry*.

[68] Forman-Hoffman V., Philibert R. A. (2006). Lower TSH and higher T4 levels are associated with current depressive syndrome in young adults. *Acta Psychiatrica Scandinavica*.

[69] Lee S., Oh S. S., Park E. C., Jang S. I. (2019). Sex differences in the association between thyroid-stimulating hormone levels and depressive symptoms among the general population with normal free T4 levels. *Journal of Affective Disorders*.

[70] Gillette G. M., Garbutt J. C., Quade D. E. (1989). TSH response to TRH in depression with and without panic attacks. *American Journal of Psychiatry*.

[71] Altuntaş S. Ç, Hocaoğlu Ç (2021). Effects of chronic suppression or oversuppression of thyroid-stimulating hormone on psychological symptoms and sleep quality in patients with differentiated thyroid cancer. *Hormone and Metabolic Research*.

[72] Kletzien H., Macdonald C. L., Orne J. (2018). Comparison between patient-perceived voice changes and quantitative voice measures in the first postoperative year after thyroidectomy: a secondary analysis of a randomized clinical trial. *JAMA Otolaryngol Head Neck Surg*.

[73] Hedman C., Djärv T., Strang P., Lundgren C. I. (2017). Effect of thyroid-related symptoms on long-term quality of life in patients with differentiated thyroid carcinoma: a population-based study in Sweden. *Thyroid*.

